# Supplementary material for: Biochemical characterisation supports a central role for Oep80 from Arabidopsis thaliana in chloroplastic β-barrel protein assembly
Source: Commun Biol. 2025 Aug 22;8:1265. doi: 10.1038/s42003-025-08689-2 (PMC12373785; doi:10.1038/s42003-025-08689-2)
Supplement: Supplementary file 1 — Supplementary information [file 42003_2025_8689_MOESM1_ESM.docx]

**Biochemical characterization supports a central role for Oep80 from *Arabidopsis thaliana* in chloroplastic β-barrel protein assembly**

Rhiannon J. Durant^1^, R. Paul Jarvis^1^ and Jani R. Bolla^1^

^1^Molecular Plant Biology, Department of Biology, University of Oxford, OX1 3RB, UK

Correspondence: jani.bolla@biology.ox.ac.uk

**Supplementary figures**


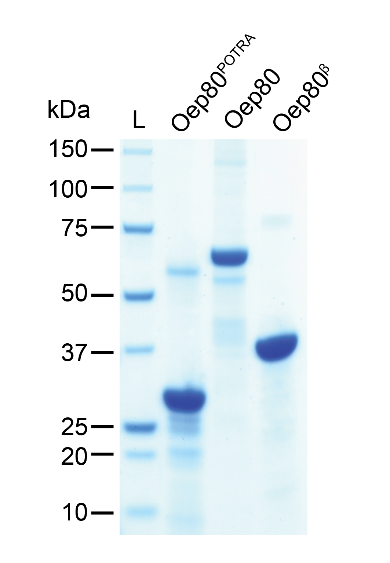


**Supplementary Figure 1. Oep80 constructs used for refolding tests showed expected masses.** SDS-PAGE gel analysis of samples from the SEC void peak of 6×His-Oep80^POTRA^ and 6×His-Oep80, and the SEC 12 mL peak of Oep80^β^-6×His. 6×His-Oep80^POTRA^ predicted mass: 29,055 Da; 6×His-Oep80 predicted mass: 66,396 Da; Oep80^β^-6×His predicted mass: 38,407 Da.


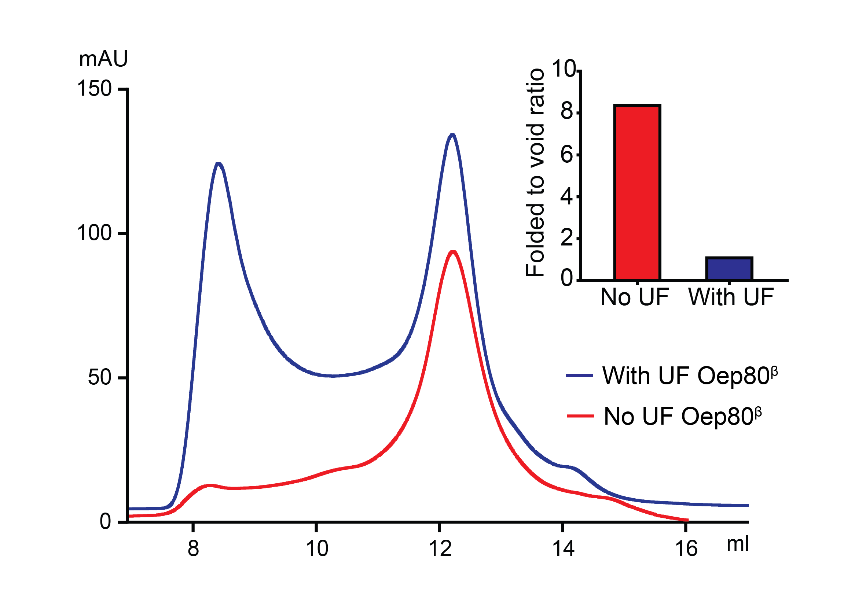


**Supplementary Figure 2. Concentration via centrifugal ultrafiltration of Oep80^β^ increases the proportion of aggregates in the sample.** Overlaid size exclusion traces of Oep80^β^-6×His after ultrafiltraton (blue) or without ultrafiltration (red; cation exchange was used to concentrate the sample instead). Inset bar chart shows ratio of the 12 mL folded peak absorbance to the 8 mL void peak absorbance. UF = ultrafiltration.


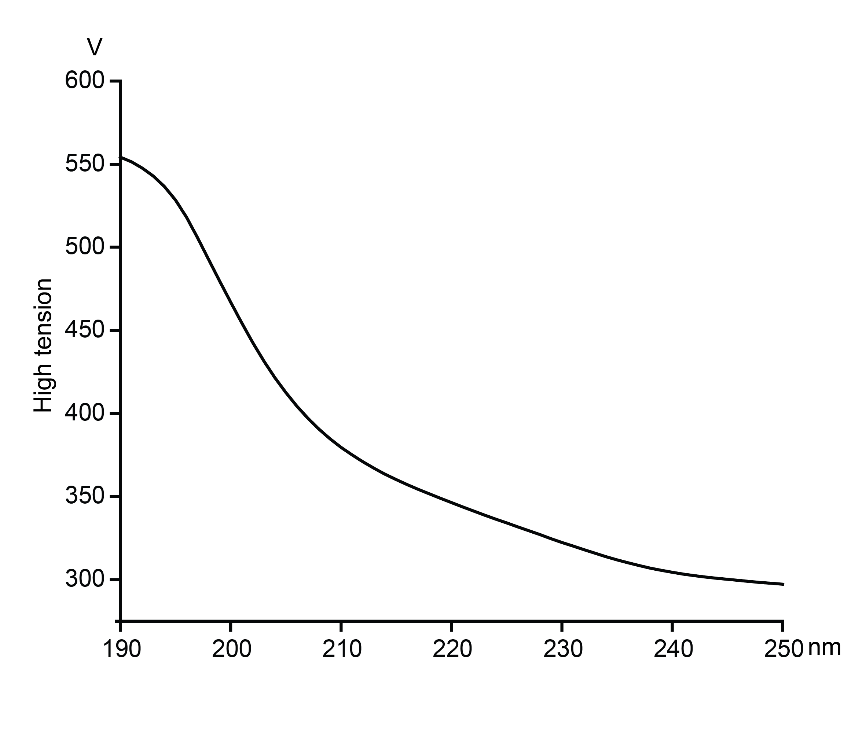


**Supplementary Figure 3. HT voltage scan.** High Tension voltage for the Oep80^β^ scan from 250 nm to 190 nm does not exceed the 600 V threshold. The HT voltage was recorded in parallel with the CD scan of Oep80^β^-6×His. Average of 10 accumulations.


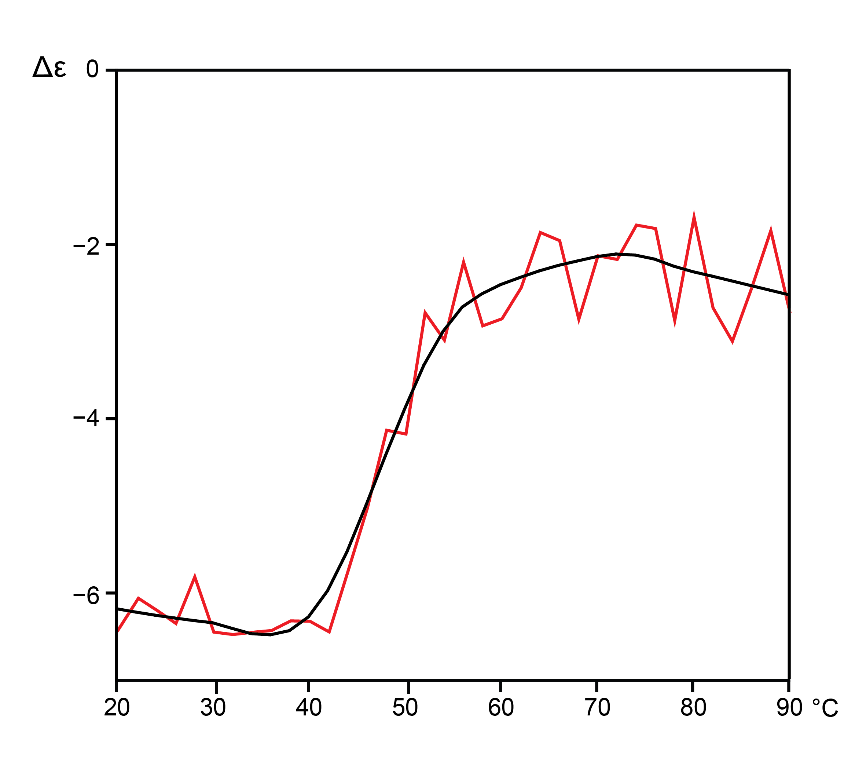


**Supplementary Figure 4. Thermal scanning CD melt curve**. The presented CD melt curve suggests that Oep80^β^ undergoes an unfolding transition with a T_m_ of around 48°C. It plots circular dichroism mean ellipticity signal against temperature. A wavelength of 218 nm was selected to monitor loss of β-sheet secondary structure. The red line shows the raw data, with scans every 2°C step. The black line shows a smoothed melting curve. The midpoint of the sharp melting transition occurs at approximately 48°C.


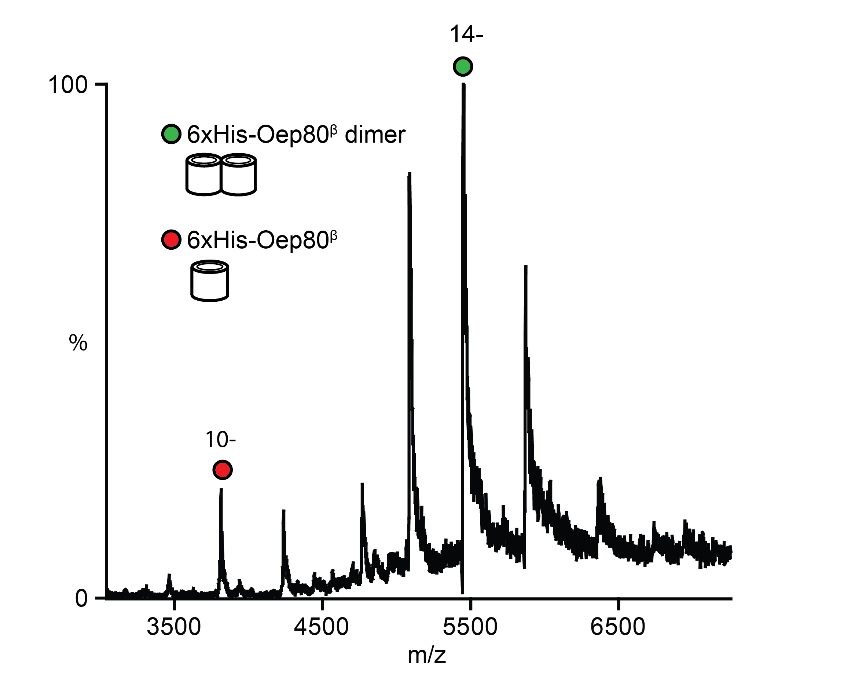


**Supplementary Figure 5. N-terminally-tagged Oep80^β^ also appears as a dimer**. Native mass spectrum of N-terminally His-tagged Oep80^β^. The spectrum indicates that this protein also appears as a dimer, like the C-terminally-tagged protein (Fig 3c). See Supplementary Table 2 for predicted and observed masses.


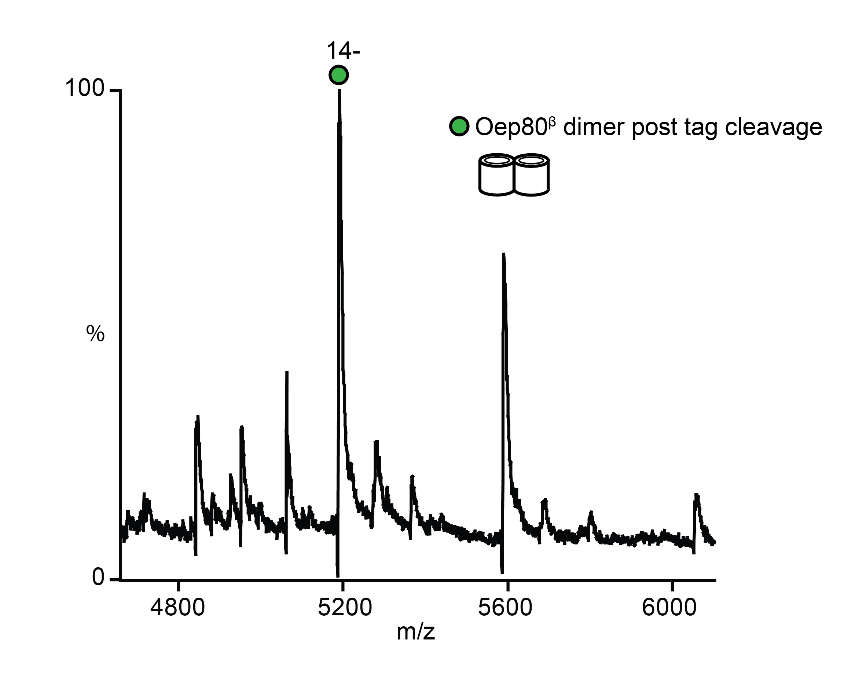


**Supplementary Figure 6. Untagged Oep80^β^ also appears as a dimer**. Native mass spectrum of 6xHis-Oep80^β^ post digestion overnight with thrombin to remove the His-tag. See Supplementary Table 2 for predicted and observed masses.


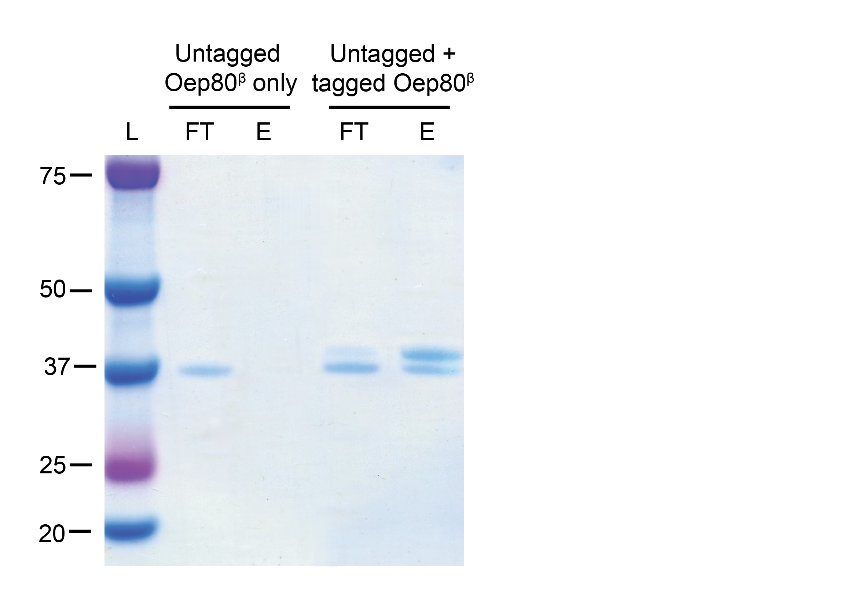


**Supplementary Figure 7. Tagged Oep80^β^ can pull-down untagged Oep80^β^.** SDS-PAGE analysis of an Ni-NTA gravity-column purification experiment performed using the indicated samples. After loading onto the column, the samples were washed extensively with 40 mM imidazole and then eluted with 300 mM imidazole. Untagged Oep80^β^ = 6×His-Oep80^β^ post-thrombin cleavage; tagged Oep80^β^ = 6×His-Oep80^β^; FT = flow-through; E = elution; L= ladder.


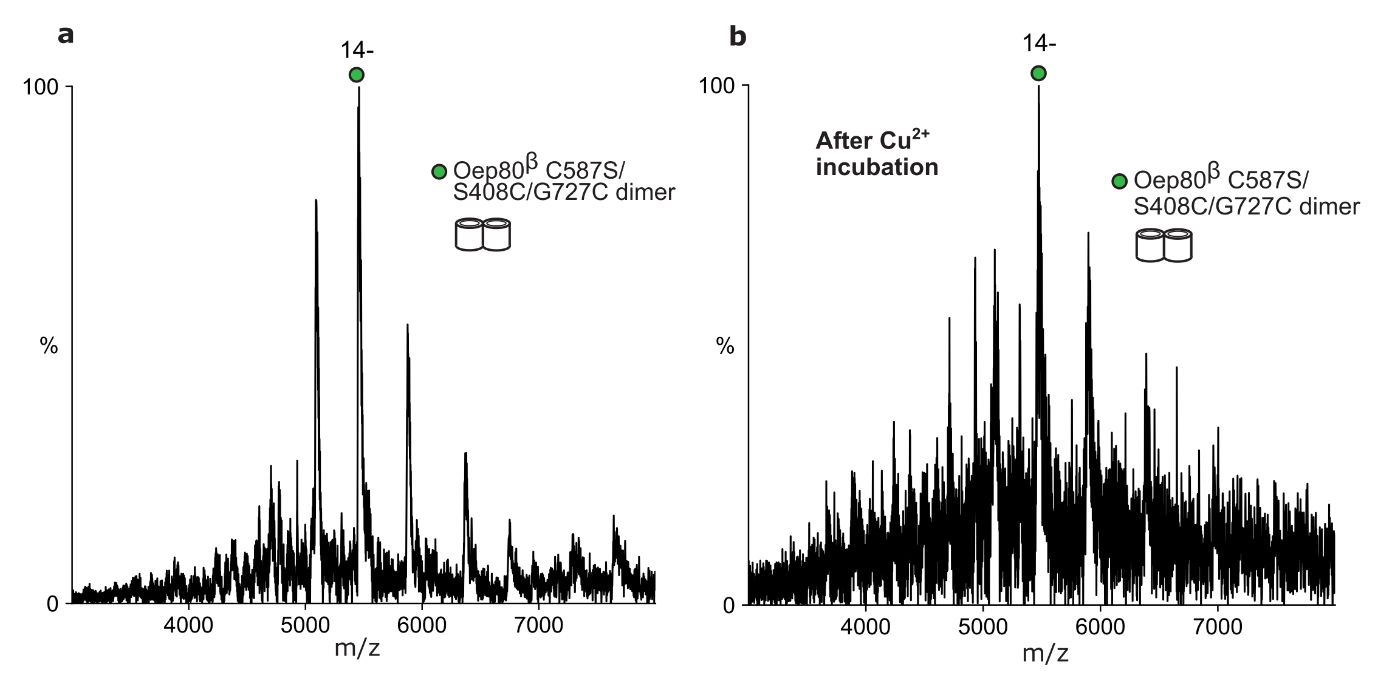


**Supplementary Figure 8. Occluding the Oep80 β_1_ strand via crosslinking does not increase monomer abundance.** Representative native mass spectra of a, Oep80^β^ C587S/S408C/G727C after purification, and b), after purification and 3 hrs of 4ºC incubation with 0.1mM CuSO_4_. See Supplementary Table 2 for predicted and observed masses.


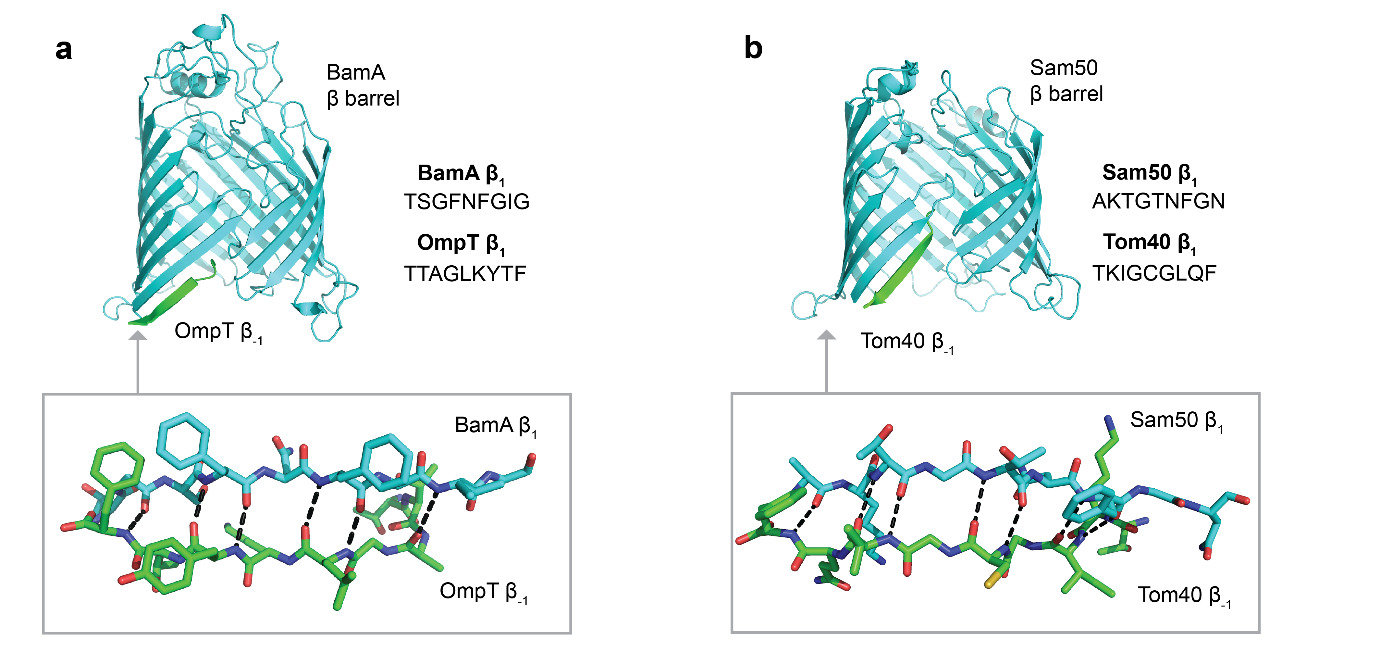


**Supplementary Figure 9. AlphaFold 3 predicts BamA and Sam50 interactions with the β-signal sequences of known substrates by β-sheet formation with the β1 strand. a**, AlphaFold 3 model of BamA interacting with OmpT β_-1_ (residues 309-317). iPTM = 0.80. **b,** AlphaFold 3 model of Sam50 interacting with Tom40 β_-1_ (residues 351-359). iPTM = 0.67. In a and b, only the barrel domains of the Omp85 proteins are shown for clarity. Black dotted lines in the insets indicate backbone N-to-O distances of 3 Å or less, i.e., likely hydrogen bonding interactions.


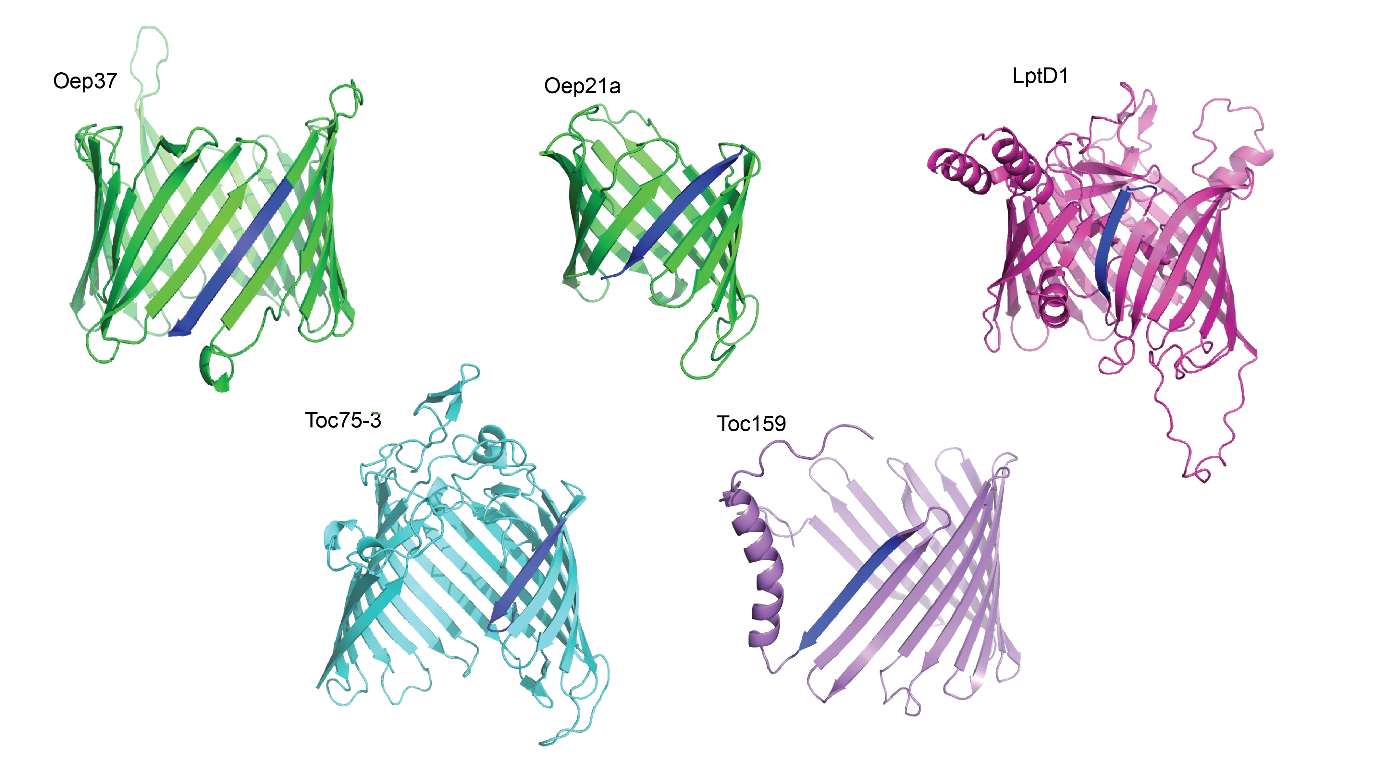


**Supplementary Figure 10. Locations of presumed β-signal sequences in representative OEPs**. Models of representative OEPs from the AlphaFold database, coloured by group assignment. Presumed β signals are coloured in dark blue. Oep37 = AF-O80565-F1-v4; Oep21a = AF-Q6ID99-F1-v4; LptD1 = AF-O80503-F1-v4; Toc75-3 = AF-Q9STE8-F1-v4; Toc159 = AF-O81283-F1-v4.


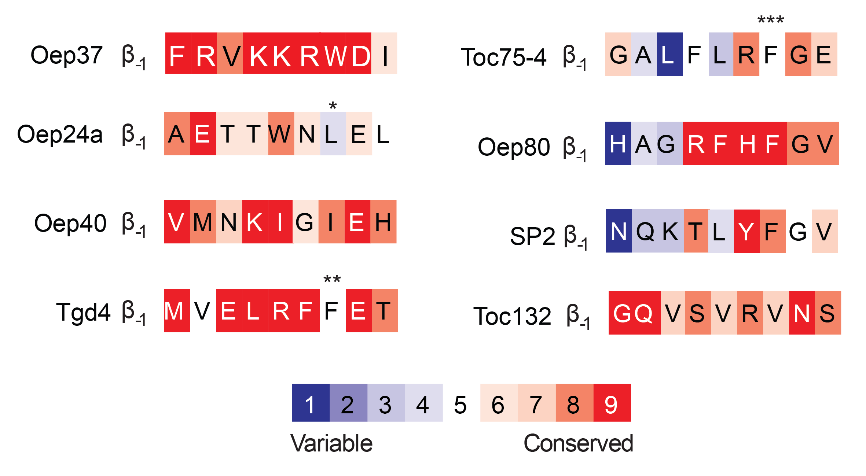


**Supplementary Figure 11. Conservation analysis on the β_-1_ strands of additional β-barrel OEPs.** Analysis conducted using ConSurf. *Majority of homologues have F at this position. ** All 150 homologues have Y or F at this position. *** Only 9 of 150 homologues differ at this position, and in all cases the residue is hydrophobic.


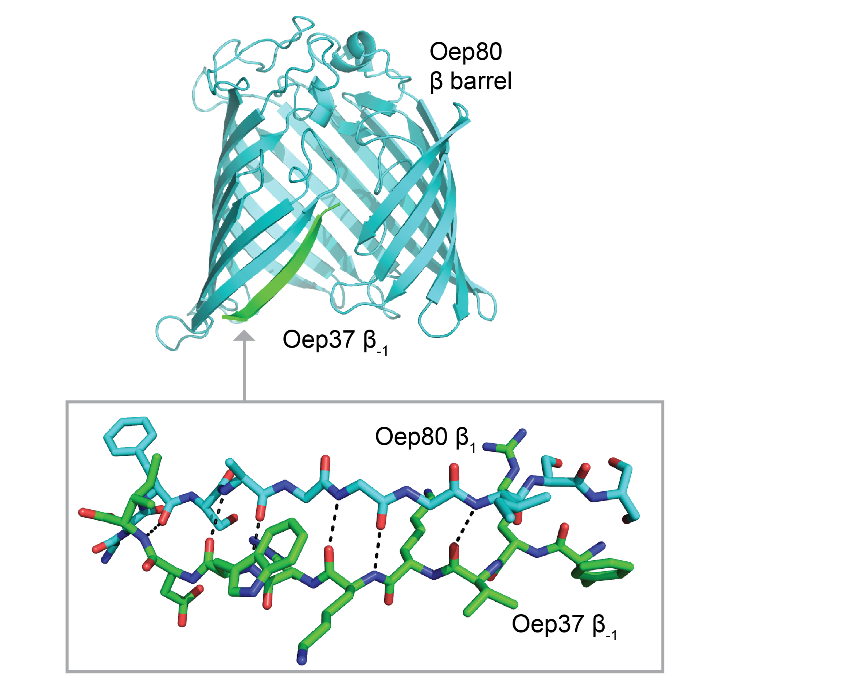


**Supplementary Figure 12. AlphaFold 3 predicts an interaction between Oep80^β^ and the β_-1_ sequence of its known substrate, Oep37**. AlphaFold 3 model of Oep80 interacting with Oep37 β_-1_ (residues 335-343). iPTM = 0.65. Only the barrel domain of Oep80 is shown for clarity. Black dotted lines in the inset indicate backbone N-to-O distances of 3 Å or less, i.e., likely hydrogen bonding interactions.


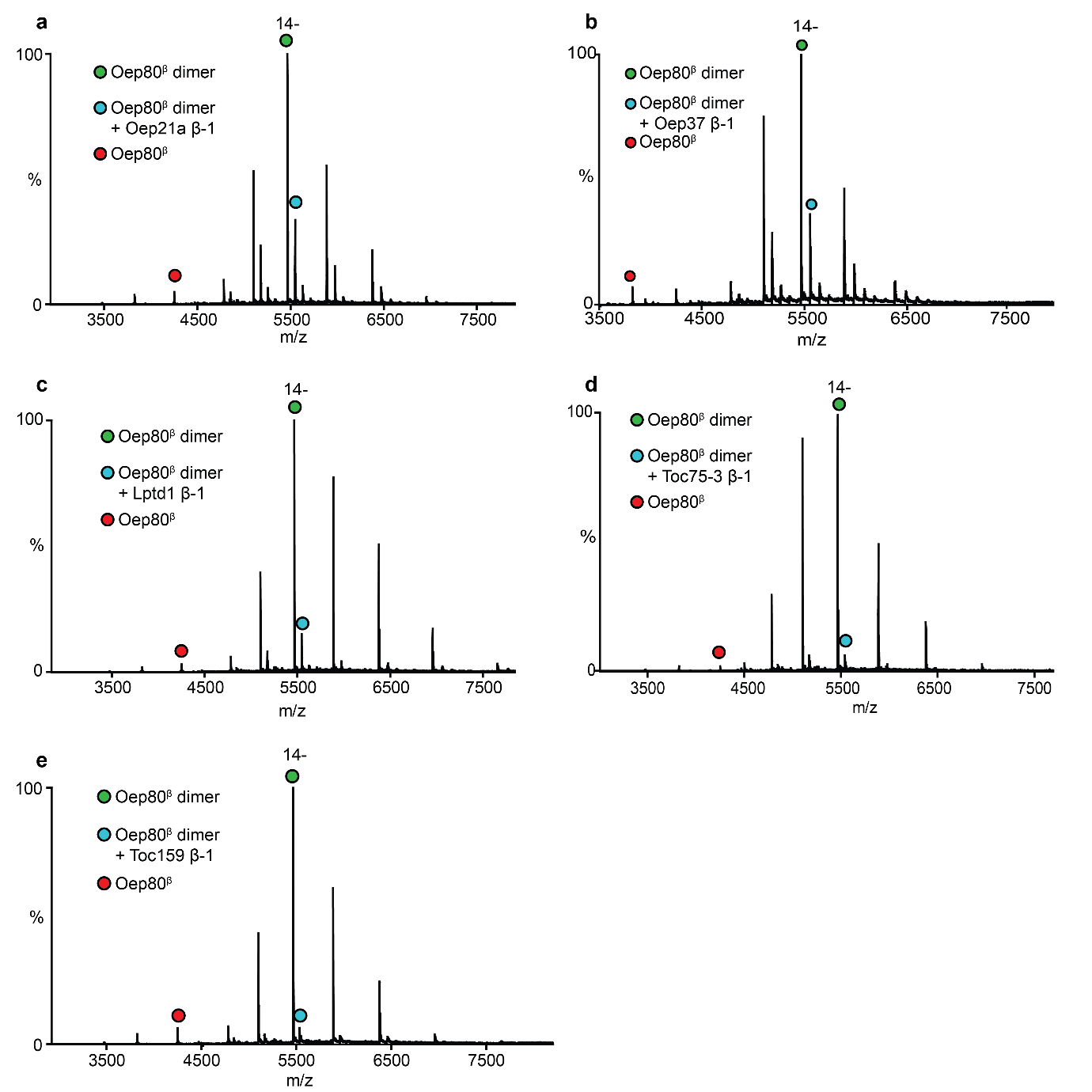


**Supplementary Figure 13. Oep80^β^ interacts with the presumed β-signal of Oep37 and Oep21a but less so with presumed β-signals of other OEPs**. Representative native mass spectra of Oep80^β^-6×His plus β_-1_ of each OEP. See Supplementary Table 4 for predicted and observed masses.


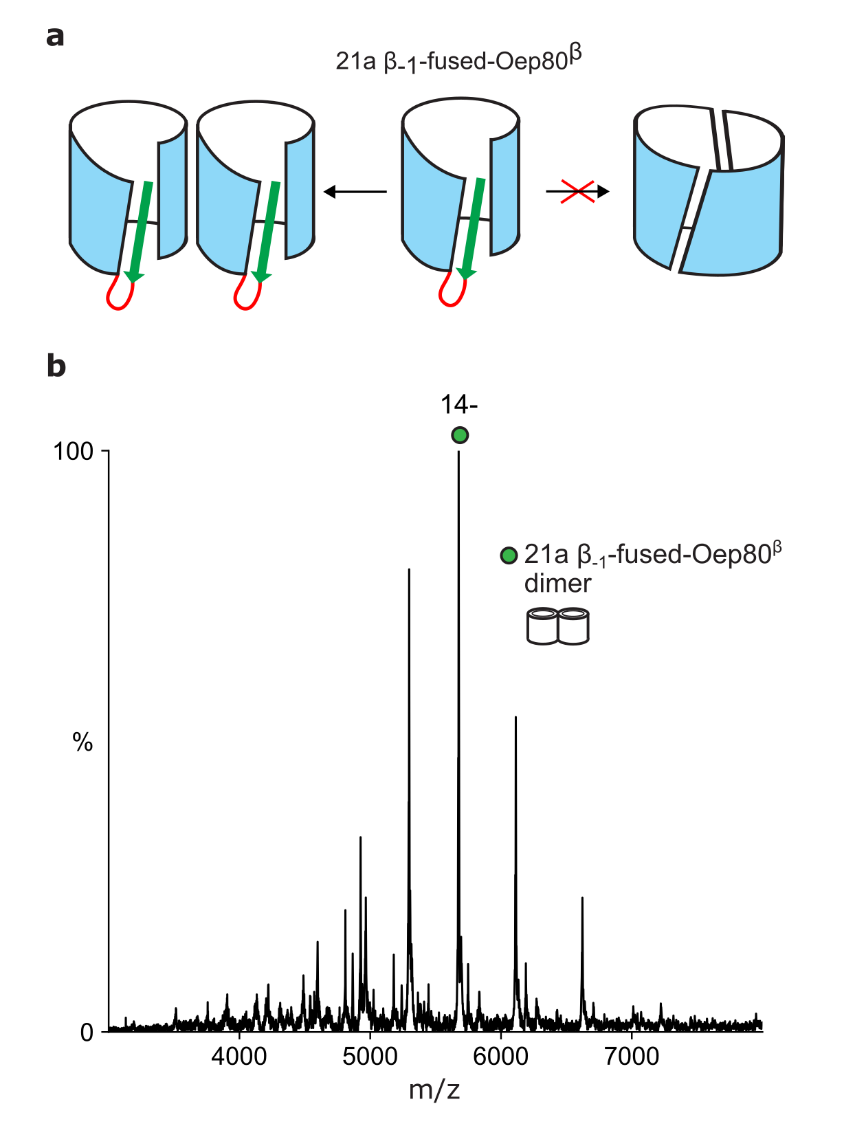


**Supplementary Figure 14. Occluding the Oep80 β_1_ strand via covalent addition of Oep21a β_-1_ peptide does not increase monomer abundance. a,** schematic to show how an N-terminally linked Oep21a β_–1_ peptide (green) might disrupt hybrid barrel formation. **b**, representative native mass spectrum of Oep21a β–1-fused-Oep80^β^. See Supplementary Table 2 for predicted and observed masses.


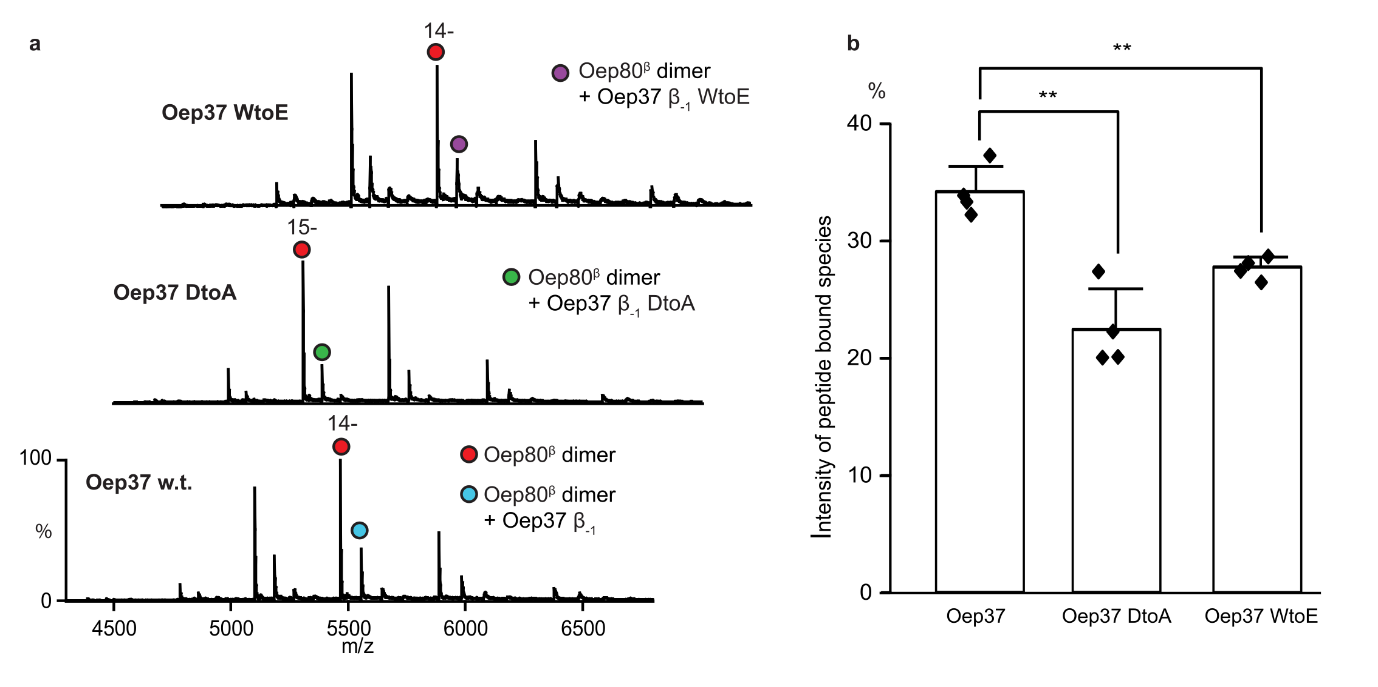


**Supplementary Figure 15. Mutations to the β_-1_ strand of Oep37 disrupts interaction with Oep80.** Oep80^β^ interacts less with the β_-1_ strand of Oep37 when its penultimate or antepenultimate residue is mutated. **a**, Native mass spectra of Oep80^β^ plus Oep37 wild-type (w.t.) peptide (FRVKKRWDI), Oep37 D-to-A peptide (FRVKKRWAI), or Oep37 W-to-E peptide (FRVKKREDI). For predicted and observed masses of species, see Supplementary Table 4. **b**, Intensity of peptide-bound Oep80^β^ relative to unbound Oep80^β^ for Oep37 wild-type peptide and the two mutants. Statistical significance was assessed using a two-tailed unpaired Student’s t-test; ** indicates p<0.01 (n=4). Error bars represent one standard deviation.


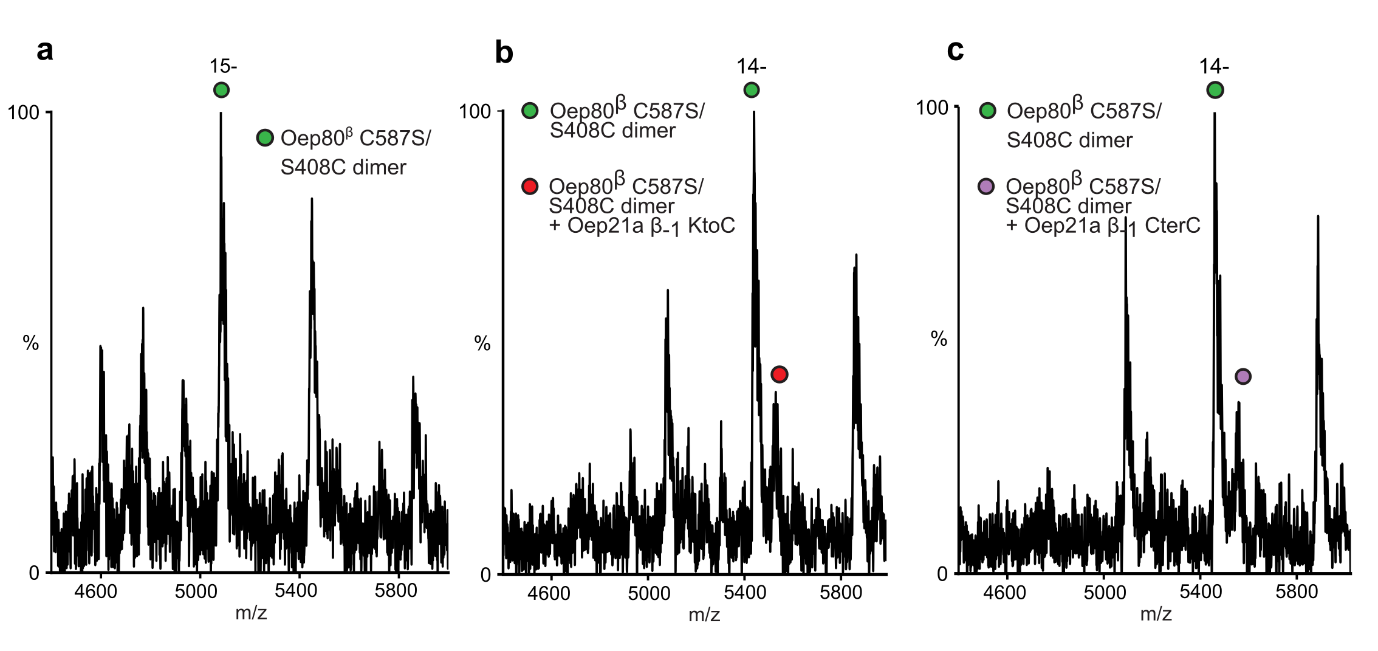


**Supplementary Figure 16. Both Oep21a β_-1_ KtoC and CterC mutants show interaction with Oep80^β^ C587S/S408C.** Native mass spectra showing: (b) Oep80^β^ **C587S/S408C** alone, (c) Oep80^β^ **C587S/S408C** + KtoC peptide, and (d) Oep80^β^ **C587S/S408C** + CterC peptide. See Supplementary Table 2 and Supplementary Table 6 for predicted and observed masses.


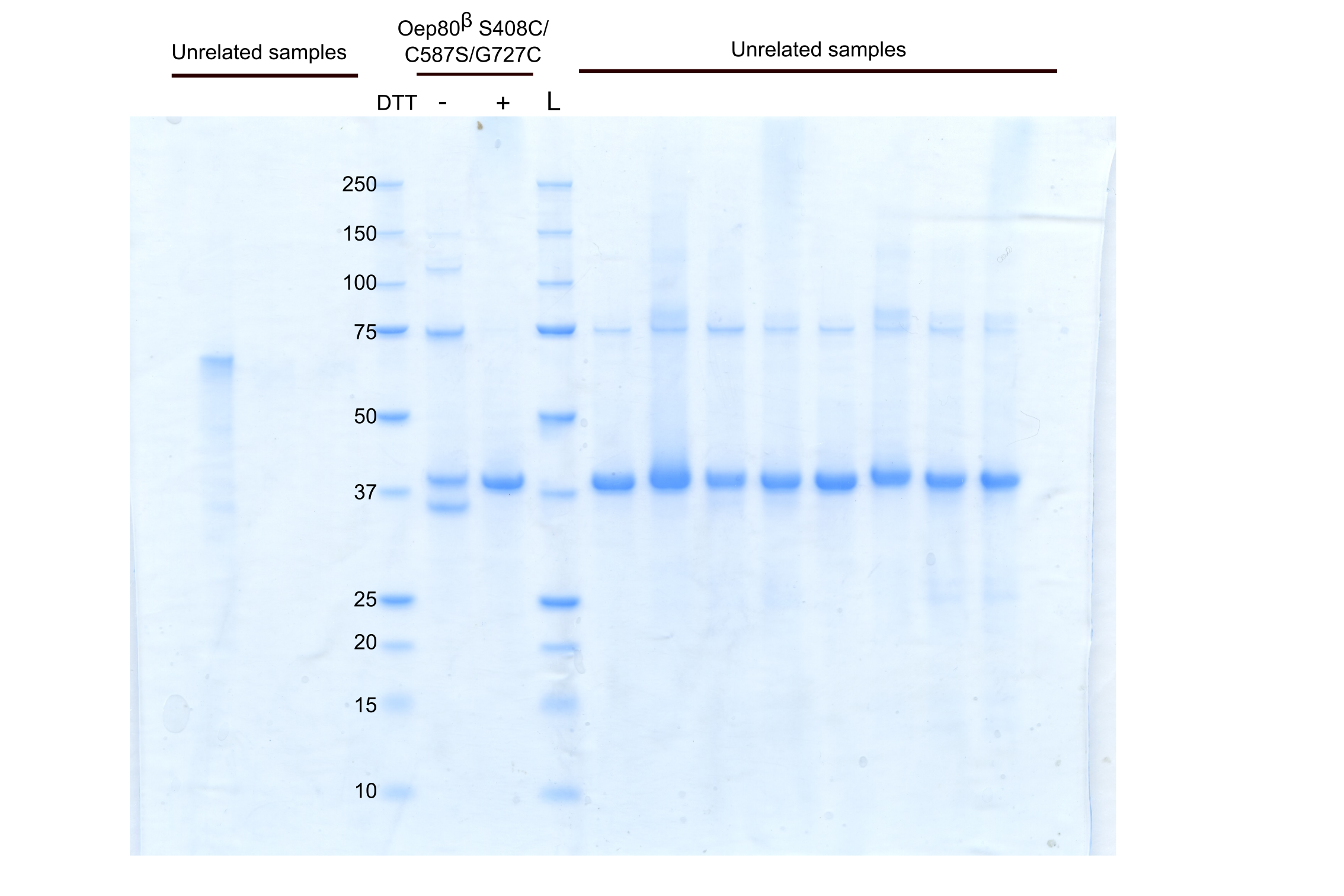


**Supplementary Figure 17. Unedited SDS-PAGE gel from Figure 3f.**


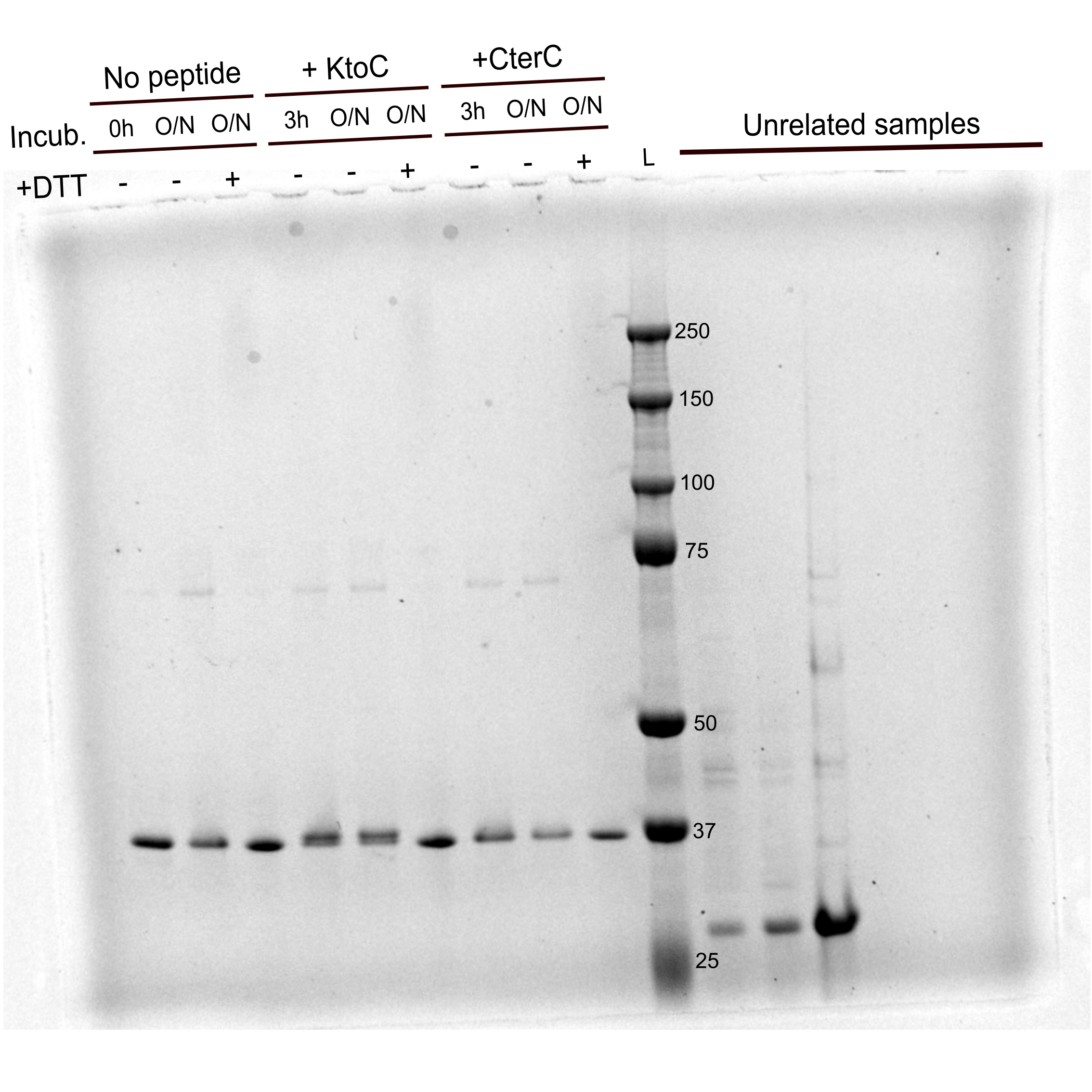


**Supplementary Figure 18. Unedited SDS-PAGE gel from from Figure 6c.** Coomassie blue stained but imaged in black and white for better focus. Recoloured to blue in Figure 6.


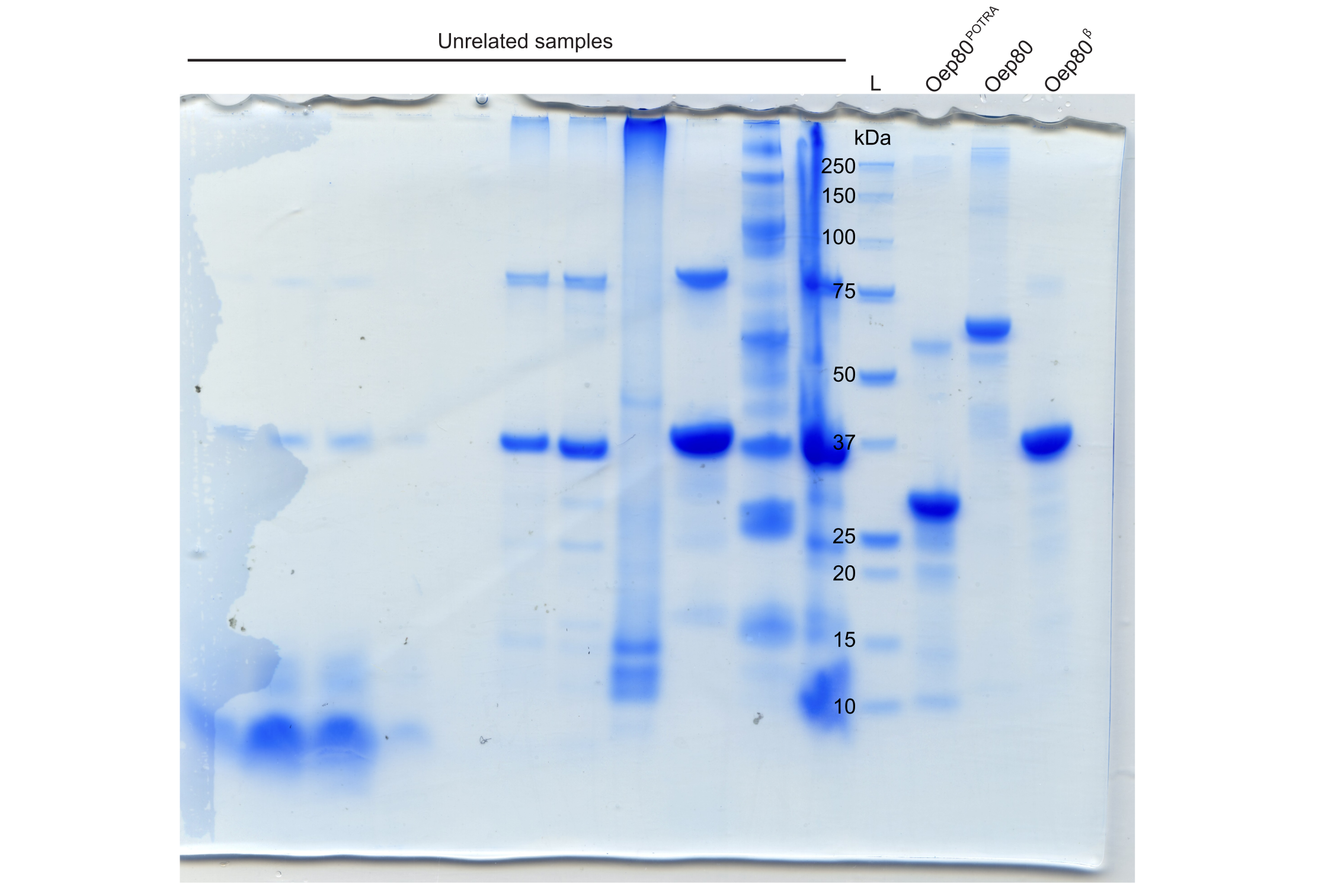


**Supplementary Figure 19. Unedited SDS-PAGE gel from Supplementary Figure 1.**


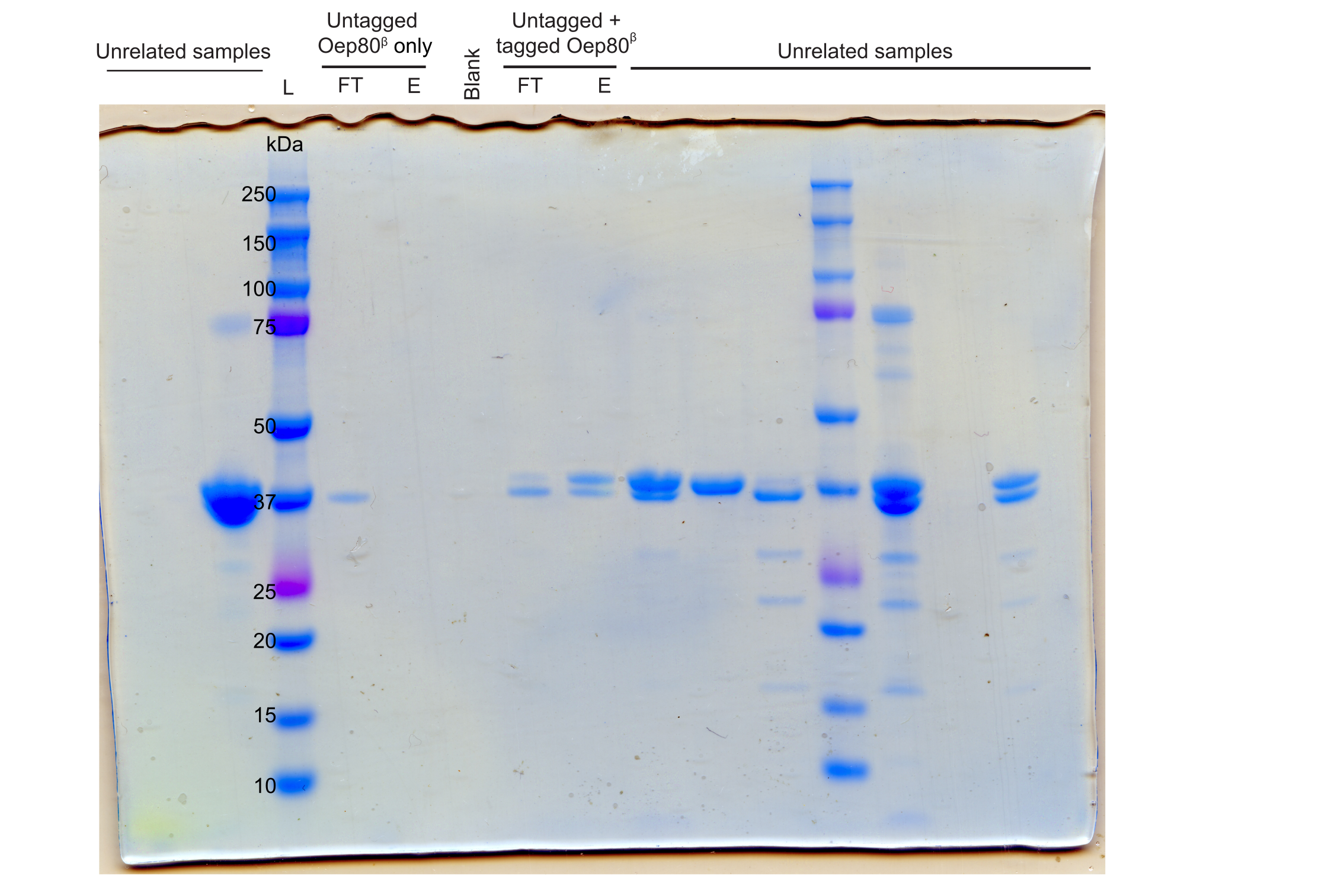


**Supplementary Figure 20. Unedited SDS-PAGE gel from Supplementary Figure 7.**

**Supplementary tables**

**Supplementary Table 1: Oep80 constructs made and tested for *E. coli* expression**

| Construct | BL21(DE3) location of expression | Other cell lines tested | Refolds without aggregation? |
| --- | --- | --- | --- |
| 6xHis-Thr-Oep80^POTRA^ | Inclusion bodies | Arctic Express: inclusion bodies only | No |
| 6xHis-Thr-Oep80 | Inclusion bodies | NA | No |
| OprM_SS-6xHis-Thr-Oep80 | Inclusion bodies | NA | No |
| Oep80-TEV-6xHis | Inclusion bodies | NA | No |
| Oep80-TEV-GFP-6xHis | Inclusion bodies | NA | No |
| 6xHis-Thr-Oep80^β^ | Inclusion bodies | NA | Yes |
| OprM-6xHis-Thr-Oep80^β^ | Did not express | BL21(DE3)PLysS: cells died | NA |
| Oep80^β^-TEV-6xHis | Inclusion bodies | NA | Yes |
| Oep80^β^-TEV-GFP-6xHis | Inclusion bodies | NA | No |
| 6xHis-Thr-Oep80^β^ C587S/S408C | Inclusion bodies | NA | Yes |
| 6xHis-Thr-Oep80^β^ C587S/S408C/G727C | Inclusion bodies | NA | Yes |
| Oep21aβ_–1_-GSGS-Oep80^β^ C587S-TEV-6xHis | Inclusion bodies | NA | Yes |

**Supplementary Table 2: Expected and observed masses for Oep80 constructs**

| Construct | Calculated mass (Da) | Observed mass (Da) | Comments | Relevant figure |
| --- | --- | --- | --- | --- |
| Oep80^β^-6xHis monomer | 38406.69 | 38253.36±1.95 | Loss of N-terminal methionine | Fig. 3c |
| Oep80^β^-6xHis dimer | 76813.38 | 76519.63±2.91 | Loss of N-terminal methionine on both monomers | Fig. 3c |
| 6xHis-Oep80^β^ monomer | 38213.64 | 38115.56±72 |  | Supp. Fig. 5 |
| 6xHis-Oep80^β^ dimer | 76427.28 | 76258.96±19.59 |  | Supp. Fig. 5 |
| Post thrombin cleavage 6xHis-Oep80^β^ dimer | 72663.18 | 72675.32±20.05 |  | Supp. Fig. 6 |
| 6xHis-Oep80 | 66396.13 | NA |  | Supp. Fig. 1 |
| 6xHis-Oep80^POTRA^ | 29055.22 | NA |  | Supp. Fig. 1 |
| 6xHis-Oep80^β^ C587S/S408C dimer | 76427.28 | 76259.21±35.64 |  | Supp. Fig. 16 |
| 6xHis-Oep80^β^ C587S/S408C /G727C dimer | 76519.44 | 76373.1±44.28 |  | Supp. Fig. 8 |
| Oep21aβ_–1_-fused-Oep80^β^-6xHis dimer | 79622.34 | 79452.14±3.27 |  | Supp. Fig. 9 |

**Supplementary Table 3: presumed β-signal peptides from all *A.thaliana* outer envelope proteins compared with β-signal for BAM and SAM**


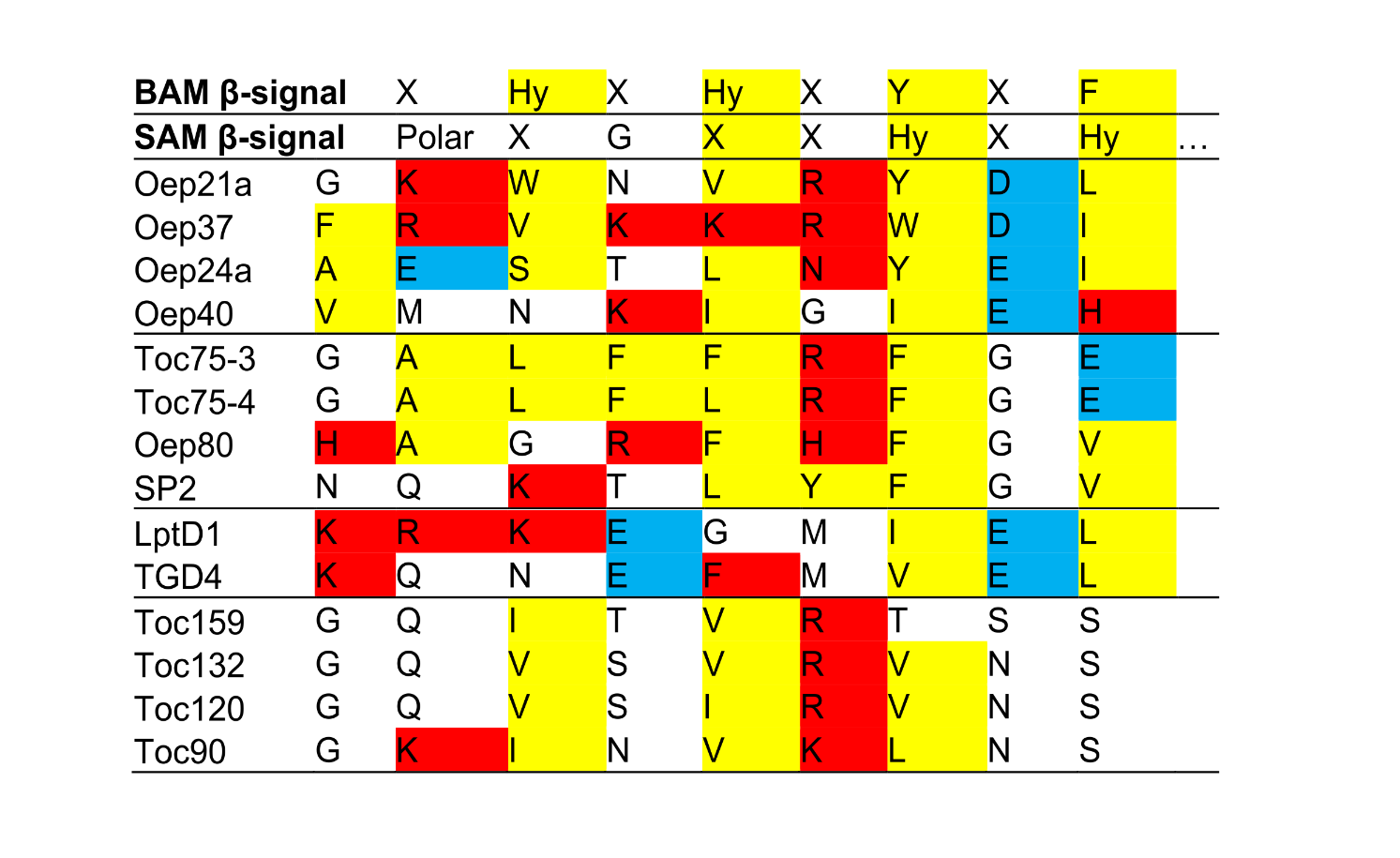


Yellow represents aromatic or hydrophobic residues, blue represents acidic residues and red represents basic residues. Hy = Hydrophobic residue.

**Supplementary Table 4: Expected and observed masses for peptide bound Oep80^β^ dimer**

| Peptide added to Oep80^β^-6xHis | Peptide mass (Da) | Predicted mass* (Da) | Observed mass (Da) | Relevant figure |
| --- | --- | --- | --- | --- |
| Oep21a β_-1_ | 1150.3 | 77671.56 | 77669.62±1.36 | Fig. 5 |
| Oep21a β_-1_ D-to-A | 1106.29 | 77627.55 | 77625.84±2.80 | Fig. 5b |
| Ooep21a β_-1_ D-to-E | 1164.32 | 77685.58 | 77684.91±4.98 | Fig. 5b |
| Oep37 β_-1_ | 1247.5 | 77768.76 | 77788.90±5.12 | Fig. 5, Supp. Fig. 11 |
| Oep37 β_-1_ D-to-A | 1203.5 | 77724.76 | 77725.69±3.07 | Supp. Fig. 11 |
| Oep37 β_-1_ W-to-E | 1190.41 | 77711.67 | 77712.01±1.87 | Supp. Fig. 11 |
| Lptd β_-1_ | 1103.35 | 77624.61 | 77624.33±2.92 | Fig. 5a |
| Toc75 β_-1_ | 1043.19 | 77564.45 | 77562.47±2.10 | Fig. 5a |
| Toc159 β_-1_ | 948.04 | 77469.3 | 77469.26±2.92 | Fig. 5a |

*Predicted mass based on observed mass of Oep80^β^-6xHis dimer alone via native MS, plus calculated mass of peptide

**Supplementary Table 5: Hydrophobicity score and net charge at pH 8 for all substrates’ β_-1_ peptides**

| Peptide | Hydropathy (GRAVY) | Charge at pH 8 |
| --- | --- | --- |
| Oep24a | -0.38 | -2 |
| Oep21a | -1.11 | 0.99 |
| Oep37 | -1.08 | 2.99 |
| Oep40 | 0.07 | 0.03 |
| Lptd1 | -1.06 | 0.99 |
| Tgd4 | -0.58 | -1 |
| Toc75-3 | 0.58 | 0 |
| Oep80 | -0.01 | 1.07 |
| SP2 | -0.28 | 0.99 |
| Toc159 | -0.3 | 0.99 |
| Toc132 | -0.1 | 0.99 |
| Oep21a_DtoA | -0.52 | 1.99 |
| Oep21a_DtoE | -1.11 | 0.99 |
| Oep37a_DtoA | -0.49 | 3.99 |
| Oep37a_WtoE | -1.37 | 1.99 |

**Supplementary Table 6: Expected and observed masses for peptide bound Oep80^β^ C587S/S408C dimer**

| **Peptide added to Oep80^β^-6xHis** | **Peptide mass (Da)** | **Predicted mass* (Da)** | **Observed mass (Da)** | **Relevant figure** |
| --- | --- | --- | --- | --- |
| Oep21a β_-1_ KtoC | 1125.27 | 77,384.48 | 77401.97±44.15 | Supp. Fig. 16 |
| Oep21a β_-1_ CterC | 1253.44 | 77,512.65 | 77523.2±65.36 | Supp. Fig. 16 |

*Predicted mass based on observed mass of Oep80^β^ C587S/S408C dimer alone via native MS, plus calculated mass of peptides

**Supplementary Table 7: Key resources and materials used in this study**

| Category | Reagent or resource | Source | Identifier |
| --- | --- | --- | --- |
| Bacterial strains | BL21 (DE3) | New England Biolabs | Cat# C2527I |
| Bacterial strains | BL21(DE3)pLysS | Invitrogen™ | Cat# C606010 |
| Bacterial strains | ArcticExpress (DE3) | Agilient | Cat# 230192 |
| Chemicals, Peptides, and Recombinant Proteins | In-Fusion® Snap Assembly Master Mix | Takara | Cat# 638947 |
| Chemicals, Peptides, and Recombinant Proteins | n-Dodecyl-β-D-Maltopyranoside (DDM) | CliniSciences Ltd | NA |
| Chemicals, Peptides, and Recombinant Proteins | Tetraethylene Glycol Monooctyl Ether (C8E4) | Thermofisher | Cat# A65524 |
| Chemicals, Peptides, and Recombinant Proteins | n-Octylglucoside (OG) | Thermo Scientific | Cat# J67390.06 |
| Chemicals, Peptides, and Recombinant Proteins | n-Dodecyl-N,N-Dimethylamine-N-Oxide (LDAO) | Anatrace | Cat# D360 |
| Chemicals, Peptides, and Recombinant Proteins | Ammonium acetate | Sigma Aldrich | Cat# A2706 |
| Chemicals, Peptides, and Recombinant Proteins | L-Arginine | Sigma Aldrich | Cat# A8094 |
| Chemicals, Peptides, and Recombinant Proteins | Dithiothreitol (DTT) | Sigma Aldrich | Cat# D9779 |
| Peptides, and Recombinant Proteins | cOmplete™, Mini Protease Inhibitor Cocktail | Sigma Aldrich | Cat# 11836153001 |
| Chemicals, Peptides, and Recombinant Proteins | All β-signal peptides | Proteogenix | NA |
| Chemicals, Peptides, and Recombinant Proteins | All gene fragments | IDT | NA |
| Chemicals, Peptides, and Recombinant Proteins | Pet28a | Novagen | Cat# 69864-3 |
| Chemicals, Peptides, and Recombinant Proteins | Pet15b | Novagen | Cat# 69661 |
| **Software and Algorithms** | UniDec | Marty et al., 2015 | <http://www.unidec.chem.ox.ac.uk> |
| **Software and Algorithms** | PyMol | Schrödinger, LLC Version 3.1 | <https://www.pymol.org/#download> |
| **Software and Algorithms** | Xcalibur | Thermo Scientific | NA |
| **Other** | Ni-NTA Agarose | Qiagen | Cat # 30210 |
| **Other** | Hi-Trap Q Fast Flow column | Cytiva | Cat # GE17-5053-01 |
